# Digital Phenotypes for Early Detection of Internet Gaming Disorder in Adolescent Students: Explorative Data-Driven Study

**DOI:** 10.2196/50259

**Published:** 2024-04-29

**Authors:** Kwangsu Cho, Minah Kim, Youngeun Cho, Ji-Won Hur, Do Hyung Kim, Seonghyeon Park, Sunghyun Park, Moonyoung Jang, Chang-Gun Lee, Jun Soo Kwon

**Affiliations:** 1 3R Innovation Research Center Seoul Republic of Korea; 2 Department of Neuropsychiatry Seoul National University Hospital Seoul Republic of Korea; 3 Department of Psychiatry Seoul National University College of Medicine Seoul Republic of Korea; 4 Department of Artificial Intelligence Hanyang University Ansan Republic of Korea; 5 School of Psychology Korea University Seoul Republic of Korea; 6 Department of Computer Science and Engineering Seoul National University Seoul Republic of Korea

**Keywords:** adolescents, digital biomarkers, digital phenotyping, digital psychiatry, early detection, IGD, internet gaming disorder, pediatric psychiatry, proactive medicine, secondary school, universal screening

## Abstract

**Background:**

Limited awareness, social stigma, and access to mental health professionals hinder early detection and intervention of internet gaming disorder (IGD), which has emerged as a significant concern among young individuals. Prevalence estimates vary between 0.7% and 15.6%, and its recognition in the *International Classification of Diseases, 11th Revision* and *Diagnostic and Statistical Manual of Mental Disorders, 5th Edition* underscores its impact on academic functioning, social isolation, and mental health challenges.

**Objective:**

This study aimed to uncover digital phenotypes for the early detection of IGD among adolescents in learning settings. By leveraging sensor data collected from student tablets, the overarching objective is to incorporate these digital indicators into daily school activities to establish these markers as a mental health screening tool, facilitating the early identification and intervention for IGD cases.

**Methods:**

A total of 168 voluntary participants were engaged, consisting of 85 students with IGD and 83 students without IGD. There were 53% (89/168) female and 47% (79/168) male individuals, all within the age range of 13-14 years. The individual students learned their Korean literature and mathematics lessons on their personal tablets, with sensor data being automatically collected. Multiple regression with bootstrapping and multivariate ANOVA were used, prioritizing interpretability over predictability, for cross-validation purposes.

**Results:**

A negative correlation between IGD Scale (IGDS) scores and learning outcomes emerged (*r*_166_=–0.15; *P*=.047), suggesting that higher IGDS scores were associated with lower learning outcomes. Multiple regression identified 5 key indicators linked to IGD, explaining 23% of the IGDS score variance: stroke acceleration (β=.33; *P*<.001), time interval between keys (β=–0.26; *P*=.01), word spacing (β=–0.25; *P*<.001), deletion (β=–0.24; *P*<.001), and horizontal length of strokes (β=0.21; *P*=.02). Multivariate ANOVA cross-validated these findings, revealing significant differences in digital phenotypes between potential IGD and non-IGD groups. The average effect size, measured by Cohen *d*, across the indicators was 0.40, indicating a moderate effect. Notable distinctions included faster stroke acceleration (Cohen *d*=0.68; *P*=<.001), reduced word spacing (Cohen *d*=.57; *P*=<.001), decreased deletion behavior (Cohen *d*=0.33; *P*=.04), and longer horizontal strokes (Cohen *d*=0.34; *P*=.03) in students with potential IGD compared to their counterparts without IGD.

**Conclusions:**

The aggregated findings show a negative correlation between IGD and learning performance, highlighting the effectiveness of digital markers in detecting IGD. This underscores the importance of digital phenotyping in advancing mental health care within educational settings. As schools adopt a 1-device-per-student framework, digital phenotyping emerges as a promising early detection method for IGD. This shift could transform clinical approaches from reactive to proactive measures.

## Introduction

### Overview

Internet gaming disorder (IGD) is characterized by persistent and repetitive engagement in online gaming. Studies consistently show that individuals with IGD experience dysfunctions in academic functioning, executive functioning, and inhibitory control, which are crucial aspects for adolescent students [1-5]. Moreover, adolescents with IGD tend to have impaired self-esteem, interpersonal relationships, and daily functioning [6]. Consequently, IGD is included in Section III of the *Diagnostic and Statistical Manual of Mental Disorders, 5th Edition, Text Revision* (*DSM-5-TR*) [7], pending formal inclusion as a disorder after further investigation. In 2019, the World Health Organization recognized IGD as a mental disorder [8].

IGD usually emerges in early adolescence [9] and significantly impacts adolescents worldwide, particularly those in secondary schools [10]. Its prevalence estimates range between 0.7% and 15.6%, with South Korea reporting around 5.9% of affected adolescents [11]. In response, the French government enacted legislation in July 2018 prohibiting the use of smartphones in schools for students from kindergarten to ninth grade. Additionally, approximately 77% of US public schools prohibit cell phone use in classrooms [12], and Korean schools either collect students’ smartphones or require them to turn off their devices when entering classrooms [13].

Therefore, early detection and intervention in at-risk young individuals affected by IGD are crucial due to these associations because early identification makes it possible to provide appropriate care for adolescents [14-17]. However, many cases are undiagnosed and untreated due to several factors, including limited awareness of IGD as a mental disorder, social stigma, and limited access to mental health professionals [18].

Digital phenotyping has not currently been developed for IGD cases, although it is promising for evaluating, diagnosing, predicting, and monitoring mental illnesses [19]. This innovative approach involves the collection and analysis of data from various digital sources, such as smartphones, wearables, and tablets, to assess an individual’s mental health and behaviors. It encompasses passive sensor data collection, including keystrokes, screen interactions, GPS location, social media use, voice tone, and physical activity. Some studies have even captured emotional states through keyboard and touch-stroking activities [20], with associations between keyboarding and various types of emotions identified [21,22]. Moreover, passive data, such as geolocation and accelerometer information, have proven successful in classifying negative symptoms in psychotic disorders [20,23]. Leveraging these sensor data, a handful of studies suggest the potential of digital phenotyping for screening IGD.

Digital phenotyping could play a pivotal role in complementing present clinical approaches by offering a proactive and continuous method of monitoring mental health for an early IGD detection role in several ways.

Unlike symptom-driven practices that rely on noticeable manifestations for patients or caregivers, digital phenotyping can detect early indicators that may not be immediately apparent in traditional assessments. By proactively identifying potential IGD concerns before they become overtly symptomatic, it supports a proactive approach to mental health care. This shift from reactive to proactive strategies aligns with the goal of early intervention and prevention of IGD. Additionally, in contrast to traditional methods, which rely on one-on-one interviews or 1-time questionnaires that may lack reliability due to social biases and limited truthful responses, digital phenotyping allows for the continuous, objective, and nonintrusive collection and analysis of adolescents’ mental health patterns. This approach is free from temporal or spatial constraints and offers near–real-time, unbiased data collection [8-10]. As emphasized by Torous et al [24], leveraging adolescents’ device engagement may enhance its acceptability and accessibility for monitoring IGD and other mental health conditions, proving cost-effective and efficient compared to traditional clinic visits. Moreover, identifying potential signs of IGD through personal devices enables early recognition, even in individuals unaware of meeting diagnostic criteria. This approach may aid in developing self-directed interventions, providing immediate mental health feedback, and facilitating timely treatment.

Digital phenotyping may play a role as a universal mental health screening tool, especially in school settings, assessing all children and adolescents irrespective of visible symptoms. Unlike the traditional practices that rely on observable symptoms noted by teachers and caregivers, the digital phenotyping approach may identify subtler indicators [19,25], enabling early detection of mental health issues [26] while students use their devices in schools. Then, subsequent diagnostic assessments can be used upon identification of concerns to make early intervention strategies [26].

The paradigm shift toward digital education in schools (eg, 1 device per student) may spur digital phenotyping methods for universal mental health screening, representing an unprecedented opportunity to apply digital phenotyping for adolescent mental health care. This is particularly evident in the context of the COVID-19 pandemic, where the ubiquity of smart devices among students has become a fundamental element of their classroom engagement. In certain countries, such as South Korea, all individual students from 3rd to 12th grade have been equipped with smart devices for both in-person classroom use and remote classroom use, initiating the gradual replacement of traditional textbooks from 2025 [27].

### Privacy and Data Security

The research, which focused on nonclinical adolescent students grappling with mental health challenges, adopted a restricted yet meaningful data collection approach. Ensuring the security and privacy of data constituted a top priority in this study. Adherence to protocols overseen by the institutional review board and compliance with legal regulations pertaining to privacy, anonymization, and data security were essential components within public school settings under government supervision. Every aspect of data collection, storage, and processing within the cloud environments, automatically managed by the Dr. Simon module of the focuspang artificial intelligence system [28], met the requisite ethical and legal compliance standards.

This unified consensus underscored the critical importance of protecting student information throughout the data collection process without interfering with teaching and learning activities.

Consequently, data collection in these settings was restricted to minimize the acquisition of information typically gathered in clinical settings, such as birth date, race, socioeconomic status, explicit content involving words, facial expressions, voice recordings, and GPS details.

### This Study

This data-driven study aimed to identify digital phenotypes for early detection of potential IGD among adolescent students in schools. Using sensor data from student tablet devices, the goal is to seamlessly integrate these digital markers into daily school routines, enabling timely identification and intervention for potential IGD cases. Emphasizing the need for seamless integration into adolescents’ daily routines in schools, the study for future mental health care aims to facilitate the timely identification of potential IGD cases within the academic framework.

The exploration of leveraging these devices for mental health care, specifically targeting IGD, forms the core objective of this study. Sensor data were gathered from student tablets while they learned Korean literature and mathematics using the tablets to investigate the viability of integrating digital phenotyping as a means of early detection.

From the screening or early detection point of view, this study underscored the differentiation between primary and secondary tests for detecting IGD. Primary tests for screening prioritize sensitivity to inclusively capture individuals with IGD, whereas secondary tests focus on specificity to accurately identify those without the disorder. Screening tests serve as a gauge for disease probability and do not offer definitive diagnoses, necessitating further evaluation through subsequent diagnostic procedures for individuals with positive results [26].

It should be noted that in this initial exploratory phase, we opted for a statistical approach instead of machine learning, prioritizing interpretability rather than predictability for future research [29]. Linear regression models can provide clear insights into the ways variables affect outcomes, enabling the drawing of significant conclusions. They act as crucial initial steps in distinguishing the digital phenotyping variations between individuals with and without IGD.

This study can make several novel contributions. Initially, the study breaks new ground by pioneering the identification of digital markers linked to IGD. This identification process has the potential to significantly contribute to early detection and prompt intervention for IGD concerns. Such contributions are crucial in preventing the escalation of IGD, especially considering that many cases remain undiagnosed and untreated. Various factors, including limited awareness of IGD as a mental disorder, social stigma, and restricted access to mental health professionals, contribute to the untreated nature of many cases, making early detection and intervention paramount.

Additionally, with the digital transformation of schools, this study may extend traditional limited clinical contributions within school settings and provide psychiatrists with a more comprehensive understanding of how IGD manifests in the context of school environments. This contextual insight can inform tailored interventions and support strategies.

In addition, this study’s focus on digital phenotyping as a tool for mental health assessment introduces a novel approach in psychiatry. The emphasis on seamlessly integrating digital markers into daily school routines showcases a practical approach to monitoring mental health. This integration could offer a continuous and nonintrusive method for tracking behavioral patterns, providing valuable insights for psychiatrists. If successful, it could pave the way for the integration of digital phenotyping into routine psychiatric assessments for a broader range of mental health conditions.

In summary, the research can contribute valuable insights and methods for early detection, intervention, and monitoring of IGD, thereby advancing psychiatry and offering practical tools for mental health care professionals.

## Methods

### Ethical Considerations

Our research protocol obtained approval from the Seoul Metropolitan Office of Education, ensuring compliance with Korean privacy and data security regulations. Additionally, it was approved by Korea University Institutional Review Board (KUIRB-2023-0159-01), aligning with the principles of the Helsinki Declaration. Before their involvement, comprehensive information about the research purpose, methods, and data collection was provided to students and their parents or legal guardians to obtain their informed consent. According to guidelines, the data collected did not include information that could potentially identify or discriminate against participants, such as birth date, ethnicity, income, or social status. Finally, as a token of appreciation for their participation, a book gift certificate worth ₩30,000 (about US $23) was provided to their parents or legal guardians.

### Participants and Their Recruitment

Between September and October 2023, middle school students in Seoul, Republic of Korea, were recruited through an official website approved by the Seoul Metropolitan Office of Education, South Korea. Initially, 935 students voluntarily participated in the study. Exclusions were made for participants with neurodevelopmental conditions, central nervous system disorders, significant medical issues affecting psychological symptoms, or an inability to use this study’s tablet computers.

After 8 (0.9%) of the 935 participants dropped out due to technical problems, the final number of participants was 927, and their average age was approximately 13 years, falling within the 13- to 14-year-old range. A total of 53% (89/168) of the participants were female, while 48% (79/168) were male.

Using the IGD Scale–Short-Form (IGDS9-SF) [30] for screening purposes, of the 927 participants, 85 (9.2%) were identified to be in the potential IGD group, while 842 (90.8%) were in the non-IGD group. To ensure balance for analysis, 83 participants without IGD were randomly selected from the pool. This approach aimed to mitigate potential biases introduced by uneven group distributions, providing a more robust foundation for our analysis. Among the total 168 participants, 89 (53%) were female, and 79 (47%) were male (Table 1).

**Table 1:** Demographic information of participants with their internet gaming disorder (IGD) scores.

Group and demographics		Participants, n (%)		IGD score, mean (SD)
**Non-IGD (n=83)**				
	Female	56 (68)	3.6 (6.8)	
	Male	27 (33)	8.1 (9.1)	
	Total	83 (100)	5.3 (8.0)	
**Potential IGD (n=85)**				
	Female	33 (39)	22.7 (4.6)	
	Male	52 (61)	23.8 (4.3)	
	Total	85 (100)	23.4 (4.4)	
**Total (n=168)**				
	Female	89 (53)	8.7 (11.2)	
	Male	79 (47)	16.1 (11.4)	
	Total	168 (100)	12.2 (11.8)	

### Procedure

Before the main session, the individual participants were individually provided with tablet computers designated for studying Korean literature and mathematics. They practiced the system interface and found no usability or technical difficulties.

The main session consisted of two 45-minute segments separated by a 15-minute break where the participants were engaged in learning tasks while sensor data were collected in the background. In the first segment, they were individually asked to respond to 20 questions in Korean literature and, in the second, 20 questions in math. Their answers to 40 questions were used as their learning performance scores.

The participants were free to use their on-screen keyboard and touchscreens to respond to the questions. Although there was no time limit, all the questions were answered during the segments. The data gathering system was designed to efficiently oversee and orchestrate each step of the learning activities, including the presentation of questions and capturing participants’ responses. Simultaneously, this system seamlessly recorded passive sensor data from the tablet, ensuring the collection of additional information related to user interaction and behaviors during the learning sessions. This comprehensive approach allowed for the simultaneous tracking of both academic engagement and the corresponding sensor data.

Following the main session, the participants were asked to fill out the questionnaires, including the IGDS9-SF [30].

### IGD Scale and Screening Criterion

The IGD Scale (IGDS), introduced by Lemmens et al [31], served as a screening tool for IGD, encompassing 9 subfactors aligned with the provisional IGD criteria of the *Diagnostic and Statistical Manual of Mental Disorders, 5th Edition* (*DSM-5*). Of the 2 versions of the scale, a 27-item and a 9-item version, this research used the Korean-adapted 9-item version of the scale, IGDS9-SF, validated to have reliability comparable to the original [32]. Each item assessed experiences over the preceding year on a Likert-type scale ranging from 0 to 5 points, yielding a total score range of 0-45. The internal consistency of this study, measured by Cronbach α, yielded a value of .84. A comprehensive review of 21 studies using the IGDS9-SF [30] reported that the scale demonstrated satisfactory internal consistency, robust criterion validity, and consistency across sex and age groups. While previous research [33] commonly used a diagnostic threshold of 21, this study opted for a lower threshold of 18, representing a 10% reduction. This adjustment came at the potential expense of a slightly increased type I error rate [26]. Monacis et al [33] demonstrated that using a cutoff score of 21, the sensitivity was 0.860 and the specificity was 0.861. When using a cutoff score of 18, the sensitivity was 0.818, and the specificity was 0.887.

### Sensor Data Collection

While the participants were engaged in their learning tasks for middle-school mathematics and Korean literature on the given tablet PCs, digital marker data were collected from 2 passive sensors, the on-screen keyboard sensor and the touchscreen sensor, of the tablet computer. Students placed their tablets on the desktop for their typing and keyboarding, deliberately minimizing any potential noises that could arise from device movements. The device was a Samsung Galaxy Tab S7 FE with Android 11 that was identical to the one the participants used in their regular classes. Data collection occurred continuously in the background at a rate of 24 Hz, equivalent to 42.67 milliseconds, using a built-in data collection module called Dr. Simon while students engaged in learning sessions on tablets. The Dr. Simon module, integrated into the focuspang artificial intelligence system, autonomously managed all data collection and learning activities [28]. This module used various sensors, including eye-tracking, voice, stylus pen, touch, keyboarding, and other smart sensors. The research measures encompassed factors such as the frequency of key presses, angles, speed, acceleration, length, duration, path, and pressure.

### Data Analysis for Interpretability over Predictability

#### Z-Score Transformation

The original measurement units of the indicators, which included self-rating, frequency, duration in milliseconds, length in pixels, pressure force, and ratio, were transformed using *z*-scores so that each indicator had a mean of 0 and SD of 1. As a result of the *z*-score transformation, indicators derived from different distributions could be compared.

#### Combined Analysis of Multiple Regression and Multivariate ANOVA

Multiple regression examines relationships between multiple predictors and a single outcome, whereas multivariate ANOVA (MANOVA) addresses multiple outcome variables at once. Together, they support a comprehensive analysis of the relationships between multiple predictors and outcomes simultaneously. Additionally, both techniques assist in adjusting for confounding variables: multiple regression controls potential confounders in independent and dependent variable relationships, while MANOVA evaluates the overall impact of independent variables on multiple dependent variables, accounting for their intercorrelations.

#### Bootstrapping Procedure

Bootstrapping, a statistical resampling technique, repeatedly samples with replacement from observed data to estimate the sampling distribution of a statistic. It is a method for robust estimates and inferences without relying on strict assumptions about the data distribution. A common approach to finding key indicators or measurements was to conduct multiple regression with a stepwise procedure. However, stepwise regression was regarded as unreliable and inadequate [34,35]. Due to this, multiple regression using a bootstrap approach was applied in this study. Using bootstrapping, 1000 sample sets were selected using the Mersenne Twisyer random number generator [34]. Furthermore, stratified sampling [36] was used to maintain the subgroup proportions of the sex types (female vs male) and IGD groups (potential IGD vs non-IGD).

## Results

### IGD and Learning Outcomes

A negative correlation was found between the scores on the IGDS and the learning performance scores on a 40-question learning assessment, indicated by *r*_166_=–0.15, with a significance level of *P*<.05. This finding underscores an inverse relationship: higher IGDS scores were associated with lower academic performance.

### Multiple Regression for Identification of Digital Phenotypes

This research used both multiple regression and MANOVA methods together to strengthen interpretative robustness. Initially, multiple regression was applied to examine how individual phenotypes relate to IGD scores. Subsequently, MANOVA was used to investigate how the classification into IGD groups might impact various digital phenotypes, allowing for a broader assessment of these connections. The use of both methods serves as a form of cross-validation. When the results from both multiple regression and MANOVA align, it bolsters the consistency of the findings, reinforcing the established relationships identified through diverse analyses.

First, initial multiple regression models were built to pinpoint indicators that effectively identify IGD. Additionally, bootstrapping was used to generate a sufficient data set even with limited data, enabling the estimation of a population’s mean. The pool of potential indicators comprised 38 candidates, encompassing various aspects of keyboard usage. This included the frequencies of specific keys, such as delete, space, backspace, keyboard switch, enter, and shift. Additionally, the pool incorporated metrics related to stroke behaviors, such as frequencies, means, or SDs of total length, horizontal length, vertical length, duration, pressure, number of points, horizontal direction, vertical direction, horizontal size, vertical size, speed, and acceleration. Further metrics involved means and SDs of keypress-to-keypress duration, pause duration before pressing enter, and release-to-press duration, among others. The assessment also considered the number of correct answers and incorrect responses.

After addressing multicollinearity issues, the final regression model selected 5 indicators (Table 2). The regression yielded statistically significant results (*F*_5, 162_=9.63; mean square error=2.09; *P*<.001), indicating that these indicators collectively accounted for *R*=0.47, *r*²=23% of the variance in IGDS.

**Table 2:** Correlation and multiple regression results between the Internet Gaming Disorder Scale and its indicators.

Variable			Correlation results						Multiple regression results					
		X1		X2	X3	X4	X5	β^a^			*t* test (*df*=162)		*P* value^b^	
**X1. Stroke acceleration**										0.33		3.81		<.001
	*r*	1		0.25	–0.38	–0.27	0.52							
	*P* value	—^c^		.001	<.001	<.001	<.001							
**X2. Time interval between keys**										–0.26		–3.18		.01
	*r*	0.25		1	–0.26	–0.53	0.19							
	*P* value	.001		—	<.001	<.001	.01							
**X3. Word spacing**										–0.25		–3.28		<.001
	*r*	–0.38		–0.26	1	0.19	–0.18							
	*P* value	<.001		<.001	—	.01	.02							
**X4. Deletion**										–0.24		–2.89		<.001
	*r*	–0.27		–0.53	0.19	1	–0.24							
	*P* value	<.001		<.001	.01	—	.002							
**X5. Horizontal length of strokes**										0.21		2.59		.02
	*r*	0.52		0.19	–0.18	–0.24	1							
	*P* value	<.001		.01	.02	.002	—							

^a^β: standardized regression coefficients.

^b^All zero-order correlations are significant (*P*<.05).

^c^Not applicable.

**Figure 1:**
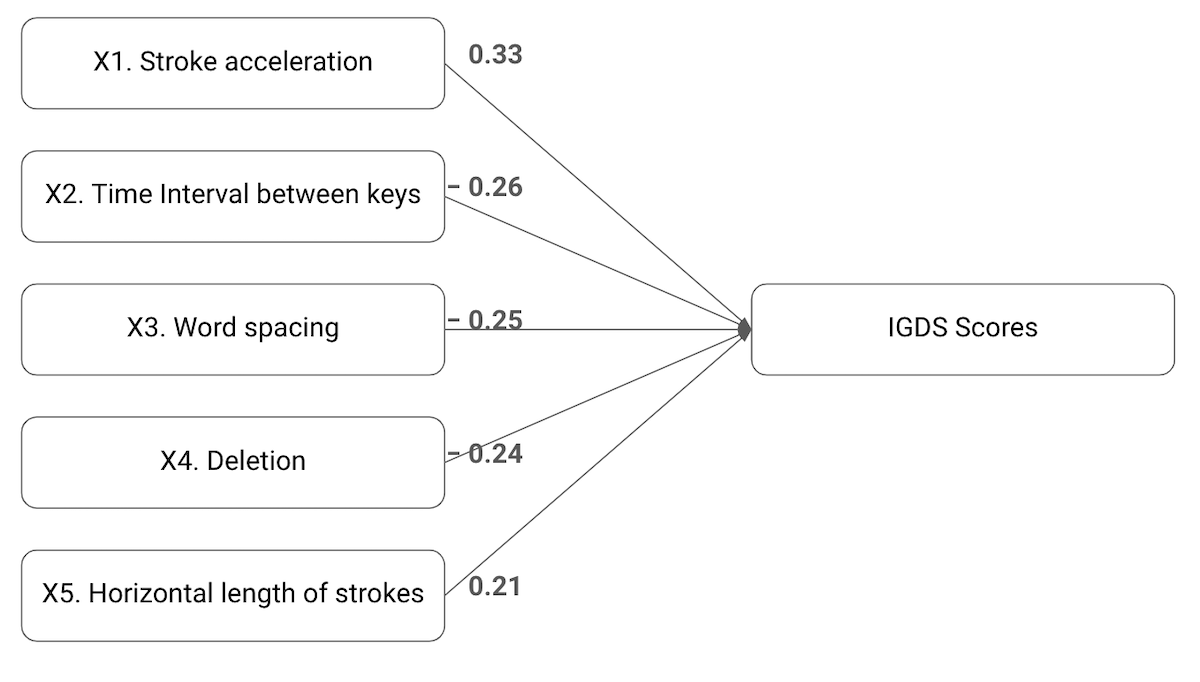
Multiple regression model based on digital phenotypes. IGDS: Internet Gaming Disorder Scale.

As shown in Table 2 and Figure 1, the IGDS scores, the dependent measure, were positively associated with the first indicator: stroke acceleration (X1). Stroke acceleration (X1) was linked to higher IGDS scores. The stroke acceleration was the speed change rate while stroking on the touch screen. When this speed increased by 1, the IGDS score increased by 0.33, assuming all other factors remained constant. Conversely, all the other indicators—time interval between keys (X2), word spacing (X3), deletion (X4), and horizontal length of strokes (X5)—showed negative associations with the IGDS scores. The time interval between keys measures how quickly the participant typed, with faster typing tending to indicate higher IGDS scores. Word spacing refers to how much space there is between letters, words, or sentences while typing. A decrease in word spacing was associated with higher IGDS scores. Deletion represents removing characters during typing. For every 1-unit increase in deletion frequency, there was an expected decrease of 0.24 units in IGDS scores, meaning lower deletion frequency is related to lower IGDS scores. Lastly, an increase in the horizontal length of strokes indicated higher IGDS scores.

In summary, the β coefficients, serving as effect sizes, offer insights into the direction and strength of relationships between each independent variable (X1-X5) and the dependent variable, the IGDS scores. Notably, all independent variables exhibit statistically significant relationships with the IGDS scores. The magnitude of the β coefficients indicates moderate relationships, with larger absolute values signifying stronger effects.

### MANOVA, Cross-Validation Through Group Comparison

To cross-validate the outcomes of the regression analysis, a comparison between the potential IGD and non-IGD groups was conducted following the method outlined by Cronbach and Meehl [37]. Using MANOVA as the methodological approach, the study used the groups as the independent variable to assess their influence on the 5 indicators, treated as dependent variables (Table 3).

**Table 3:** Multivariate ANOVA comparison between the internet gaming disorder (IGD) and non-IGD groups.

Dependent variable	IGD, mean (SD)	Non-IGD, mean (SD)	Sum of square (*df*)	Mean squares	*F* test (*df*=163)	*P* value	Cohen *d*
X1. Stroke acceleration	–0.28 (0.85)	0.40 (1.12)	19.32 (1)	19.32	19.51	<.001	0.68
X2. Time interval between keys	0.12 (1.04)	0.04 (0.95)	0.31 (1)	0.31	0.31	.58	0.09
X3. Word spacing	0.21 (1.01)	–0.36 (0.99)	13.67 (1)	13.67	13.65	<.001	0.57
X4. Deletion	0.11 (1.03)	–0.22 (0.95)	4.43 (1)	4.43	4.52	.04	0.33
X5. Horizontal length of strokes	–0.03 (1.03)	0.34 (1.16)	6.02 (1)	6.02	4.98	.03	0.34

The multivariate test yielded a substantial Wilks λ value of 0.799, accompanied by *F*_5,162_=8.164, with a significance level of *P*<.001. The corresponding effect size, measured through η-squared, revealed a moderate magnitude of 0.23.

Upon conducting univariate ANOVAs, noteworthy disparities (*P*<.01) surfaced between the IGD and non-IGD groups concerning stroke acceleration (X1; *P*<.001), word spacing (X3; *P*<.001), deletion (X4; *P*=.04), and horizontal length of strokes (X5; *P*=.03). Specifically, potential IGD students displayed faster stroke acceleration, lower word-spacing tendencies, decreased deletion behavior, and longer horizontal strokes compared to non-IGD students.

However, no significant difference was observed in the time interval (X2; *P*=.48) between the groups. The divergence between the outcomes of multiple regression and MANOVA could stem from their inherent methodological dissimilarities: multiple regression focuses on predicting a single dependent variable using multiple predictors, while MANOVA evaluates the collective impact of 1 or more independent variables on multiple dependent variables simultaneously. This variation in approach might lead to discrepancies in determining significance [38]. Notably, considering that multicollinearity was controlled in the multiple regression, it appears to have limited influence on these findings.

## Discussion

### Overview

The aim of this exploratory study was to find digital indicators to be used for early detection of potential IGD among adolescent students in their learning activities. The surge in digital education trends—1 device per student—in public schools may present an unprecedented avenue for deploying digital phenotyping as a universal mental health screening tool in the management of young individuals’ mental health and academic progress. Thus, digital phenotyping holds the potential to disrupt the detrimental cycle wherein school experiences and IGD mutually exacerbate each other [39,40].

### Principal Findings

An initial investigation into the connection between IGD and academic performance uncovered a negative correlation, indicating that higher IGDS scores were associated with lower learning performances.

The main analyses, the multiple regression and MANOVA procedures with bootstrapping revealed 5 markers: stroke acceleration, the time interval between keys, word spacing, deletion, and the horizontal length of strokes. These findings suggest that the identified digital markers for IGD are reliable across multiple methods and may offer valuable insights into understanding how IGD operates in a learning context, connecting clinically useful knowledge for addressing the issue of IGD in educational settings.

The identified indicators seem to suggest that adolescent students with IGD tend to engage in learning activities rapidly and roughly rather than meticulously and accurately. This type of speedy behavior characterizes these students. This aligns with previous research by Kim et al [2], who found that children displaying gaming addiction are more likely to exhibit impulsive behavior, defined as a fast reaction without thinking or conscious judgment [2]. Similarly, Şalvarlı and Griffiths [41] conducted a comprehensive review of 33 empirical studies involving a total of 18,128 participants and reported that, with 1 exception, all 32 studies showed a positive association between impulsivity and IGD.

The tendency of students with IGD to act quickly and impulsively may negatively impact their learning outcomes. Impulsive students are more prone to making errors due to the speed-accuracy trade-off theory [42], which suggests that acting quickly can lead to reduced accuracy. Several neurophysiological studies have also linked IGD to impulsivity, impaired response inhibition, and dysfunctional attentional bias [2,3]. Moreover, Lee et al [4] found that patients with IGD have impaired reading ability.

Along with the negative correlation between the number of correct answers as learning outcomes and the IGDS score, all of the digital indicators—the stroke acceleration (*r*=–0.41), the time interval between keys (*r*=–0.30), word spacing (*r*=0.42), deletion (*r*=0.16), and horizontal length of strokes (*r*=–0.29)—were significantly correlated with the number of correct answers at *P*<.05. These correlations collectively further reinforce the notion that impulsive and speedy behaviors in IGD students may impact their learning outcomes.

The study concludes by suggesting a practical application of digital phenotyping in schools, particularly due to the recent digitalization of education. Smart devices are now available in in-person classes as well as in remote learning settings [27,43]. Using these devices, digital markers for IGD can be used to diagnose and improve students’ mental health while they are in school. Digital phenotypes offer a potential solution to break the vicious cycle between learning and IGD [39,40,44,45].

### Limitations

While the findings of this study suggest the potential use of digital markers for screening IGD during learning activities in schools, it is essential to approach the results with caution, considering several limitations.

First, before accepting digital markers as direct proxies for IGD, further research and validation are imperative. The study used an inductive or data-driven approach due to the limited theoretical frameworks for digital phenotyping in mental health. Consequently, the identified indicators require scrutiny for psychiatric validity. An additional constraint in this study lies in the lack of confirmed IGD diagnoses following the early detection or screening phase. Future research should integrate a comprehensive diagnostic process to validate the presence of IGD.

Second, it is crucial to note that in this study, IGD was treated as a unitary construct. However, Wang et al [46] identified at least 2 subgroups of people with IGD, each exhibiting different brain functional connectivity patterns and distinct psychiatric symptom profiles. Therefore, future phenotypic studies need IGD as a multifaceted and complex system.

Third, the IGDS questionnaire served as the standard for recognizing digital phenotypes. However, concerns about the credibility of psychiatric disorders lacking objective measures and depending on subjective self-reporting have prompted neuroscientist Insel [47] to characterize digital phenotyping as a “new behavioral science.”

Fourth, it is crucial to test phenotypes across diverse age groups, particularly involving younger individuals. Younger individuals often display more pronounced and rapid responses to various stimuli, making them essential for understanding the initial effects of certain phenotypes or interventions. Studying phenotypes in younger age groups can offer insights into the early onset of certain traits or conditions, allowing for proactive interventions or preventive measures. This is particularly vital in fields such as health care or education, where early identification of certain phenotypes can enable tailored interventions to support healthy development or address potential issues before they escalate.

Fifth, digital phenotyping faces various challenges, with data quality standing out among them. Typically relying on data collected from smartphones or smartwatches in natural environments, these studies encounter distinct patterns due to device movements, differences among individuals, and variations within the same individual across multiple observations. This can lead to data gaps and irregularities. However, in this specific study, data maintained a high standard due to consistent collection methods with minimal missing data and disturbances. For example, placing tablets on students’ desks helped minimize disruptions from their movements. Furthermore, all participants underwent similar conditions, spending about similar time on 2 similar tasks, thereby mitigating variations between individuals and within individuals.

### Conclusions

The findings establish a correlation between IGD and academic performance, revealing significant distinctions in digital phenotypes between potential IGD and non-IGD cohorts. This highlights the potential use of specific digital markers for early identification of potential IGD students. However, it is imperative to emphasize the necessity for further research and validation to bolster the reliability of these digital markers and deepen our comprehension of the intricate nature of IGD.

Finally, the study proposes a practical application of digital phenotyping, considering the prevalence of smart devices not only in traditional classrooms but also in remote learning environments [27,47]. By leveraging these devices, digital markers for IGD can be deployed to diagnose and enhance students’ mental well-being during their time in school. Essentially, digital phenotypes present a promising avenue to disrupt the detrimental cycle between learning and IGD.

## Abbreviations

DSM-5: Diagnostic and Statistical Manual of Mental Disorders, 5th Edition
DSM-5-TR: Diagnostic and Statistical Manual of Mental Disorders, 5th Edition, Text Revision
IGD: internet gaming disorder
IGDS: Internet Gaming Disorder Scale
IGDS9-SF: Internet Gaming Disorder Scale–Short-Form
MANOVA: multivariate ANOVA
