# Correction: Digital Phenotypes for Early Detection of Internet Gaming Disorder in Adolescent Students: Explorative Data-Driven Study

**DOI:** 10.2196/60568

**Published:** 2024-06-06

**Authors:** Kwangsu Cho, Minah Kim, Youngeun Cho, Ji-Won Hur, Do Hyung Kim, Seonghyeon Park, Sunghyun Park, Moonyoung Jang, Chang-Gun Lee, Jun Soo Kwon

**Affiliations:** 1 3R Innovation Research Center Seoul Republic of Korea; 2 Department of Neuropsychiatry Seoul National University Hospital Seoul Republic of Korea; 3 Department of Psychiatry Seoul National University College of Medicine Seoul Republic of Korea; 4 Department of Artificial Intelligence Hanyang University Ansan Republic of Korea; 5 School of Psychology Korea University Seoul Republic of Korea; 6 Department of Computer Science and Engineering Seoul National University Seoul Republic of Korea

In “[Digital Phenotypes for Early Detection of Internet Gaming Disorder in Adolescent Students: Explorative Data-Driven Study]” ([JMIR Ment Health 2024;11:e50259 doi: 10.2196/50259]) the authors noted the following errors.

1. In the Results section of the Abstract, the sign in the following sentence:

*Horizontal length of strokes (β= –0.21)*

was corrected to:

*Horizontal length of strokes (β=0.21)*

2. In Figure 1, the sign of the coefficient of the Horizontal length of strokes:

*–0.21*

was corrected to:

*0.21*

3. In Table 2, the sign of the β^a^ of the Horizontal length of strokes,

*–0.21*

was corrected to:

*0.21*

4. In Table 2, the *t* test value’s sign of the Horizontal length of strokes:

*–2.59*

was corrected to:

*2.59*

5. In Table 1, the column titles:

*Potential IGD, Non-IGD*

were corrected to:

*Non-IGD, Potential IGD*

The correction will appear in the online version of the paper on the JMIR Publications website on June 6, 2024, together with the publication of this correction notice. Because this was made after submission to PubMed, PubMed Central, and other full-text repositories, the corrected article has also been resubmitted to those repositories.

